# Adult Neurogenesis in Sheep: Characterization and Contribution to Reproduction and Behavior

**DOI:** 10.3389/fnins.2017.00570

**Published:** 2017-10-23

**Authors:** Frederic Lévy, Martine Batailler, Maryse Meurisse, Martine Migaud

**Affiliations:** Institut National de la Recherche Agronomique, UMR85, Centre National de la Recherche Scientifique, UMR7247, Université F. Rabelais, IFCE, Physiologie de la Reproduction et des Comportements, Nouzilly, France

**Keywords:** hypothalamus, hippocampus, olfactory bulb, photoperiod, maternal behavior, neuroblasts, seasonal reproduction, neurogenic niche

## Abstract

Sheep have many advantages to study neurogenesis in comparison to the well-known rodent models. Their development and life expectancy are relatively long and they possess a gyrencephalic brain. Sheep are also seasonal breeders, a characteristic that allows studying the involvement of hypothalamic neurogenesis in the control of seasonal reproduction. Sheep are also able to individually recognize their conspecifics and develop selective and lasting bonds. Adult olfactory neurogenesis could be adapted to social behavior by supporting recognition of conspecifics. The present review reveals the distinctive features of the hippocampal, olfactory, and hypothalamic neurogenesis in sheep. In particular, the organization of the subventricular zone and the dynamic of neuronal maturation differs from that of rodents. In addition, we show that various physiological conditions, such as seasonal reproduction, gestation, and lactation differently modulate these three neurogenic niches. Last, we discuss recent evidence indicating that hypothalamic neurogenesis acts as an important regulator of the seasonal control of reproduction and that olfactory neurogenesis could be involved in odor processing in the context of maternal behavior.

## Introduction

A majority of invertebrates and vertebrates species show a continuous addition of neurons throughout life. Neurogenesis occurring in species with long development and life span (e.g., carnivores, ungulates, and primate) is far much less documented than in rodents. However, the timing of generation, migration, and differentiation of new neurons have been reported to differ according to lifespan and life history (Amrein et al., 2011). These differences are not surprising when one considers that neurogenesis is part of the plastic changes allowing adaptation to functional demands that differ according to species.

Sheep are an interesting species for studying neurogenesis for several reasons. Its development (puberty at 6–8 months) and its life expectancy (10–12 years) are rather long in comparison to rodents and differences in life span could influence the rate of neuronal maturation in adulthood. Sheep possess a gyrencephalic brain, a cortex with a laterally expanded folded pial surface similar to non-human and human primates, and adult neurogenesis could differ from a lissencephalic brain with a smooth cortical surface, like rodents, since major developmental differences exists between both types of brain (Fietz et al., 2010). It is hypothesized that brain expansion accompanied by gyrification and topographical complexity leads to dramatic differences in the migration and maturation rates of newborn neurons (Paredes et al., 2016). Sheep is also a seasonal breeder, unlike the majority of laboratory rodents, and these seasonal changes are under the control of the hypothalamic region. Because this region has also the capacity to produce adult-born neurons (see below), the ovine model allows one to study the role of such plasticity in the regulation of seasonal reproduction. Species such as sheep that live under different complexity of social organization and in a more natural environment than laboratory rodents would extend our knowledge of the functional significance of adult neurogenesis in social contexts (Gheusi et al., 2009). Sheep are highly social and develop selective and stable bonds. In this species, odors play a key role in individual recognition of conspecifics either in male-female or mother-young interactions (Keller and Lévy, 2012). Thus, sheep offer a unique opportunity to understand how adult olfactory neurogenesis, one of the numerous forms of brain plasticity constitutes an adaptive response to social behavior by favoring recognition of conspecifics.

**Figure 1:**
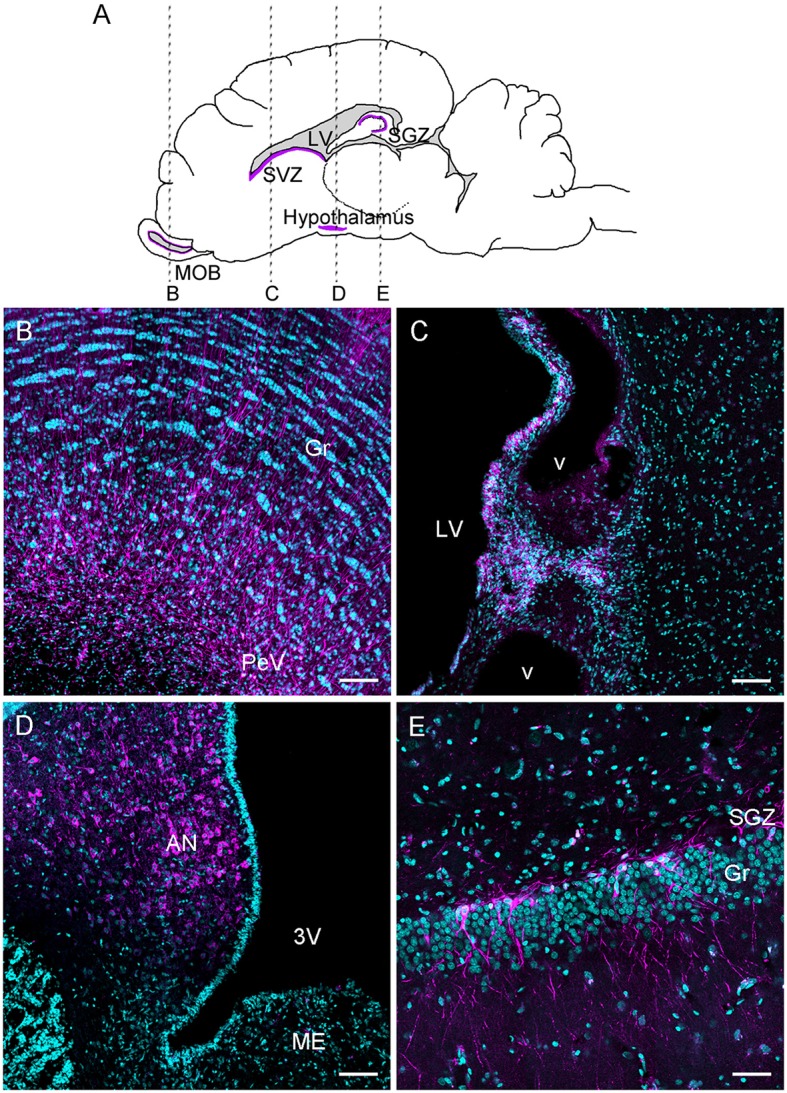
Schematic drawing of a sagittal ewe brain representing rostro-caudal levels of B, C, D, E photomicrographs **(A)**; DCX immunoreactive cells and fibers (magenta) in the main olfactory bulb **(B)**, subventricular zone **(C)**, hypothalamus **(D)**, and dentate gyrus of the hippocampus **(E)**. The sections were counterstained with Hoechst (cyan). AN, arcuate nucleus; Gr, granular layer; LV, lateral ventricle; 3V, third ventricle; ME, median eminence; PeV, periventricular zone; SGZ, subgranular zone; SVZ, subventricular zone; v, blood vessel. Scale bar: 100 μm.

The aim of this review is to point out recent findings on the characterization of olfactory, hippocampal, and hypothalamic neurogenic niches in sheep (Figure 1A). In addition, their regulation by physiological status and social interactions are considered and the possible functional relevance of these different forms of adult neurogenesis is discussed in the context of seasonal regulation and social interactions.

## Characterization of olfactory, hippocampal, and hypothalamic neurogenic niches

### Olfactory neurogenesis

#### SVZ organization and migration pathway

As in rodents and primates, adult neural stem cells in sheep are located in the subventricular zone (SVZ) and produce neurogenic and gliogenic precursors (Figure 1C). These precursors migrate toward the main olfactory bulb (MOB) forming a pathway called the rostral migratory stream (RMS; for an extended review, see Doetsch et al., 1999a; Gil-Perotin et al., 2009; Brus et al., 2010; Sawamoto et al., 2011). In a study establishing the origin of adult-born neurons in the MOB, an AAV5-eGFP was injected in the SVZ (Brus et al., 2013). AAV5-eGFP labeled cells were observed in the MOB when the injection site was located in the SVZ, but not when located posterior at the level of the corpus callosum. This result indicates a similar distribution of neural stem cells between ovine and rodents SVZ (Luskin, 1993). In addition, the SVZ is expanded to the sheep MOB because their lateral ventricle extends up to the MOB, as is the case in rabbits (Luzzati et al., 2003; Brus et al., 2013). Like rabbits and primates, chains of neuroblasts in the anterior SVZ are immersed within an astrocytic meshwork. The presence of a hypocellular layer separating chains of neuroblasts from the ependymal layer resembles the SVZ composition in bovines (Rodriguez-Perez et al., 2003) and primates (Quinones-Hinojosa et al., 2006; Gil-Perotin et al., 2009). Thus, in sheep, from the SVZ up to the MOB a migratory pathway is found along the lateral ventricle from the SVZ to the ventricle of MOB (Brus et al., 2013).

Cells, labeled by injection of an AAV5-eGFP into the SVZ, and found in the periventricular layer of the MOB show an undifferentiated phenotype at 2 months of age. They are mostly Sox2+, a marker of proliferating precursor cells and of glial-like cells; they show a simple dendritic arborization. Likewise, 1-day-old bromodeoxyuridine+ (BrdU) cells are observed in the periventricular layer of the MOB and are co-labeled with Ki-67, an endogenous mitotic marker (Kee et al., 2002). These cells are co-labeled with GFAP, which is a marker for astrocytes and neural stem/progenitor cells (Chojnacki et al., 2009), further suggesting the existence of a neurogenic niche within the MOB (Doetsch et al., 1999b; Brus et al., 2010). Similarly, neuroblasts, evidenced by DCX labeling, are also found in the periventricular layer with a round or fusiform shape and short processes, whereas deeper in the granular cell layer of the MOB, neuroblasts are more mature and display multiple processes (Figure 1B). Thus, a continuous zone of neural progenitors exists along from the lateral ventricle to the rostral extreme of the MOB ventricle. This characteristic was confirmed by Low et al.'s (2013) study and resembles the human RMS (Curtis et al., 2007). Thus, in sheep, the periventricular layer of the MOB could also constitute a pool of cells that could supply new neurons that migrate to the granular layer. However, cell proliferation rate in the MOB is much lower than in the SVZ. Interestingly, the MOB of mice (Gritti et al., 2002), macaque monkeys (Kornack and Rakic, 2001), and humans (Pagano et al., 2000; Bedard and Parent, 2004) also contains neural progenitors. The ability of stem cells isolated from the MOB to produce neurospheres is weaker than those from the SVZ, though (Gritti et al., 2002). Hence, in some species, the periventricular layer of the MOB could be neurogenic but its activity may be relatively minor.

#### Dynamics of maturation

**Figure 2:**
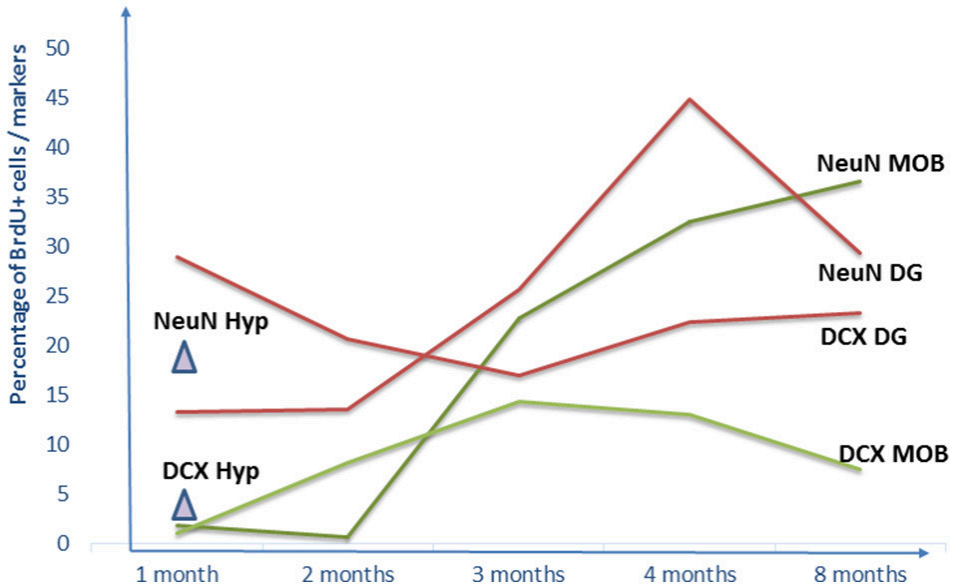
Dynamics of adult neurogenesis in the main olfactory bulb, the dentate gyrus of the hippocampus and the hypothalamus. Results are expressed as percentage of BrdU+ cells per markers for NeuN+ and DCX+ at 1, 2, 3, 4, and 8-month BrdU post-injections. No data are available after 1-month post-injection for the hypothalamus. DG, dentate gyrus; Hyp, hypothalamus; MOB, main olfactory bulb.

Although adult neurogenesis is well-conserved in mammals, some features differ between species. In addition to the organization of the SVZ, the dynamic of neurogenesis appear to vary between short-lived and long-lived mammals (Amrein, 2015). For instance, in the MOB of rodents, the far majority of newborn neurons are observed within 15 days after BrdU injections and are fully mature 15 days later (Petreanu and Alvarez-Buylla, 2002; Winner et al., 2002; Brown et al., 2003; Imayoshi et al., 2008). By contrast, in the macaque, only a very small population of BrdU positive cells is found even at 3 months post-injection in the granular cell layer (Kornack and Rakic, 2001). In female sheep, using BrdU injections in combination with maturation markers, noticeable differences in the dynamic of neuronal maturation are found in comparison to rodent adult neurogenesis (Brus et al., 2013; Figure 2). For instance, in sheep no variation of BrdU cell density is observed across time except a decrease at 8-month post-injection, suggesting a slow process of apoptosis over this period, in contrast to rodents in which half of the newborn cells die within the first month after birth (Alvarez-Buylla et al., 2001; Lemasson et al., 2005). Very few neuroblasts (BrdU+/DCX+ cells) are found at 1 month after BrdU injections in the granular layer of the sheep MOB. This population peaks at 3-month and decreases slowly up to 8 months after BrdU injections. No mature neurons (BrdU+/NeuN+ cells) are observed before 3 months post-injections and the highest proportion of new neurons is found 8 months after BrdU injections. These new cells could be activated because they express immediate early genes indicating their functional integration into the granular cell layer. In addition, a substantial proportion of immature cells, evidenced by Sox2 labeling, is found both in the periventricular and granular layers, again supporting the hypothesis of the presence of stem cells that could differentiate according to physiological challenges.

### Hippocampal neurogenesis

#### Evidence for a neurogenic niche

It is currently clear that the dentate gyrus (DG) of the hippocampus is a neurogenic niche for the majority of the mammalian species investigated so far (for a brief overview, see Vadodaria and Gage, 2014; for an extended review, see Amrein, 2015). In Merino ewes, Hawken et al. (2009) reported for the first time the existence of cell proliferation in the DG using BrdU immunohistochemistry. A more detailed study shows in Ile-de-France ewes that 1 day-old BrdU + cells are indeed present in the DG and that their density is highest in the subgranular layer (SGZ) than in the granular layer (Figure 1E; Brus et al., 2010). A confocal analysis reveals that almost the entire population of these cells also expresses Ki-67, and half of the BrdU+ cell population co-localizes with GFAP. In addition, a significant population of Sox2+ cells is present in the subgranular layer of the DG. Unexpectedly, a high percentage of these cells are found for up to 8 months (Brus et al., 2010), suggesting that a pool of presumed stem cells continues to generate new neurons over a long time, as has been reported in mice (Ninkovic and Gotz, 2007). Cell proliferation in the DG was also evidenced in another domestic breed of sheep, Romney/Suffolk ewes (Low et al., 2013), and in a feral breed of sheep (Hazlerigg et al., 2013), the Soay sheep. However, in the Soay breed, a very low rate (2 cells/mm^2^) was found in comparison to the domestic breed (30 cells/mm^2^ in Brus et al.'s (2010) study) albeit despite the dose of BrdU injected in that study being three-fold. Whether this difference is due to disparities in methodology or to breed differences remain unknown.

#### Dynamics of maturation

Similar to what is observed in the MOB, neuronal maturation in the DG takes a longer time in sheep than in rodents. In the DG of rodents, the majority of neuroblasts is observed at 7 days after BrdU injection and this population declines by 30 days (Brown et al., 2003; Kempermann et al., 2003; Steiner et al., 2004; McDonald and Wojtowicz, 2005; Suh et al., 2007). In sheep, the proportion of neuroblasts peaks at 1 month and stays fairly stable up to 8 months post-injection (Figure 2; Brus et al., 2013). A similar timing of maturation of the neuroblasts is reported in the macaque monkey (Kohler et al., 2011). As for the mature neurons, in rodents they are detected at 10 days post-injection and their number increases up to 1-month survival time (Cameron et al., 1993; Brown et al., 2003; Steiner et al., 2004; McDonald and Wojtowicz, 2005; Suh et al., 2007). In sheep, a low proportion of DCX+ or NeuN+ cells is observed at 1 month BrdU post-injection and the proportion of mature neurons peaks at 4 months survival time (Brus et al., 2013). Similarly, in primates, very few mature neurons are found at 1-month survival time and are the most numerous at 6-months survival time (Kohler et al., 2011; Sawamoto et al., 2011).

In both the MOB and the DG, <50% of the BrdU+ cells turn into mature neurons even at 8 months post-injections, in contrast to mice and rats in which the majority of BrdU+ cells become neurons (Petreanu and Alvarez-Buylla, 2002; Winner et al., 2002; Suh et al., 2007; Imayoshi et al., 2008). In summary, olfactory and hippocampal neurogenesis in sheep are characterized by delayed neuronal maturation, similar to primates (Kohler et al., 2011). However, one cannot exclude that the production of neurons by progenitor cells is reduced in comparison to rodents, as was shown in primates (Tonchev and Yamashima, 2006). Although the mechanisms underlying differences in the length of maturation between species remain to be determined, a parsimonious hypothesis could be related to differences in their life spans.

### Hypothalamic neurogenesis

#### Historic

Fifteen years ago, the neurogenic activity observed in the mammalian brain was thought to be limited to the two defined regions described above. However, numerous pieces of evidence indicate that other brain regions retained the capacity to produce adult-born neurons under physiological conditions. Indeed the existence of new neurons has been revealed in the hypothalamus (Kokoeva et al., 2005; Xu et al., 2005; Migaud et al., 2010), the amygdala (Bernier et al., 2002; Luzzati et al., 2003; Akbari et al., 2007; Ahmed et al., 2008), the striatum (Bedard et al., 2002, 2006; Emsley et al., 2005), the piriform and prefrontaly cortices (Bernier et al., 2002; Pekcec et al., 2006; Staffend et al., 2014) as well as more caudally in the substantia nigra (Zhao and Janson Lang, 2009) and the midbrain raphe nuclei (Holschbach and Lonstein, 2017).

**Figure 3:**
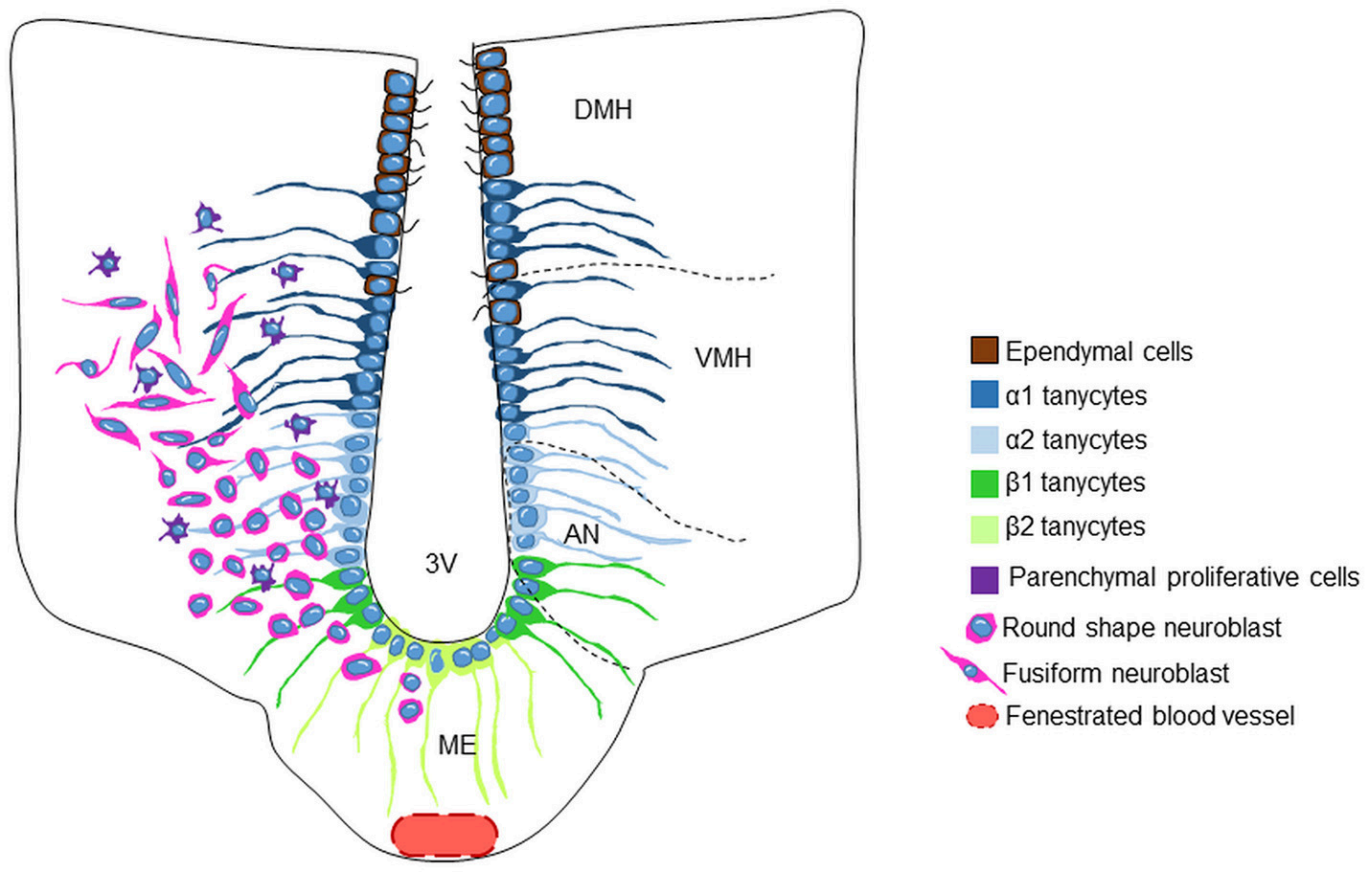
Schematic representation of the tanycytic/stem cell populations in the adult sheep hypothalamic neurogenic niche. The third ventricle (3V) wall is composed of ciliated ependymal cells and four different populations of tanycytes. The medial part of the 3V contains α1-and α2-tanycytes that contact the VMN and the ARN nuclei, respectively. The ventral part of the 3V wall (median eminence) contains β1- and β2-tanycytes, the latter residing on the floor of the 3V. β2-tanycytes are in contact with the hypothalamic-pituitary portal system through fenestrated blood vessels. The round shaped neuroblasts are located in the AN and become more fusiform when they reach the VMH. Progenitor cells are detected throughout the VMH and AN parenchyma. DMH, dorsomedial hypothalamus; VMH, ventromedial hypothalamus; AN, arcuate nucleus; 3V, 3rd ventricle; ME, median eminence.

Among these structures the hypothalamus has received much attention (reviews: Migaud et al., 2010; Lee and Blackshaw, 2012, although its level of constitutive neurogenesis seems lower than in the two well-documented neurogenic regions (Lee and Blackshaw, 2012). Constitutive hypothalamic neurogenesis has been proven to occur in many species of mammals, including mice (Kokoeva et al., 2005, 2007; Lee et al., 2012; Li et al., 2012; Werner et al., 2012; review in Lee and Blackshaw, 2012), rats (Pencea et al., 2001; Xu et al., 2005; Pérez-Martín et al., 2010), voles (Fowler et al., 2005), hamsters (Huang et al., 1998; Mohr and Sisk, 2013), and sheep (Migaud et al., 2010, 2011; Batailler et al., 2014, 2016; Figure 3), indicating that structural plasticity involving *de novo* cell genesis is an evolutionary conserved process possibly taking place in humans as well (Dahiya et al., 2011; Batailler et al., 2014). The hypothalamus is limited anteriorly and posteriorly by the optic chiasm and the mammillary bodies, respectively, whereas the optic tract constitutes its lateral perimeter. This small ventral brain region is symmetrically located between the third ventricle and contains numerous distinct nuclei (Figure 3). Constant hypothalamic neurogenic sites have been located in the subependymal zone of the third ventricle of the arcuate nucleus and the median eminence (ME; Kokoeva et al., 2005; Yuan and Arias-Carrion, 2011; Cheng, 2013). This neurogenesis process may be involved in hypothalamic regulatory mechanisms, including in the control of energy balance and in the regulation of reproductive physiology.

#### Hypothalamic cell proliferation

There are now numerous pieces of evidence indicating that new neurons can be formed outside the SGZ and the SVZ, in niches located in other structures of the adult brain. The presence of cells having incorporated the proliferation marker BrdU in the adult hypothalamus was first reported in 2001 in the rat (Pencea et al., 2001). These authors showed for the first time a proliferative activity outside the neurogenic niches and demonstrated that the number of cells in proliferation increased after brain-derived neurotrophic factor (BDNF) infusion. Few years later, a more comprehensive study, revealed the presence of neural progenitor cells (NPCs) located in the ependymal layer of the adult third ventricle, including tanycytes-derived NPCs (Xu et al., 2005). From then, it was shown that hypothalamic cell proliferation occurs constitutively without conspicuous contribution of external signals (Huang et al., 1998; Pencea et al., 2001; Fowler et al., 2002; Kokoeva et al., 2005; Xu et al., 2005; Pérez-Martín et al., 2010; Migaud et al., 2011; Mohr and Sisk, 2013) regardless of the concentration of the proliferation marker used, the route of administration, or the species being studied.

In sheep, constitutive cell proliferation in the whole hypothalamus is detected 24 h after a single intravenous (i.v.) BrdU injection (Migaud et al., 2010, 2011) and substantial proliferative activity is observed in the hypothalamus of Ile-de-France ewe (i.e., ~400 BrdU+ nuclei per hypothalamic section). BrdU+ nuclei are often seenas contacting cell pairs, which is a characteristic of recent or ongoing mitotic activity (Migaud et al., 2011). Similarly, in the male Soay sheep, dividing cells are detected in the hypothalamus following two consecutive i.v. injections of BrdU. Mitotic cells have been detected in the three distinct hypothalamic regions the ependymal cell layer, the median eminence and a region covering the tanycyte projection zone (Hazlerigg et al., 2013). More recently, the use of proliferating cell nuclear antigen (PCNA) a cell proliferation marker confirms ongoing hypothalamic new cell generation in the arcuate nucleus and the ME of adult sheep (Batailler et al., 2014). However, no BrdU+ cells are detected in the hypothalamus of female Merino sheep (Hawken et al., 2009). These inconsistencies could be attributed to the differences in the technical procedures used, including the protocol for BrdU immunodetection (e.g., different primary antisera concentrations of HCl used).

#### Fate of the hypothalamic newborn cells

The hypothalamic mitotic cells in sheep adopt a neuronal phenotype as revealed by the high number of immature neurons that express DCX (Batailler et al., 2014; Migaud et al., 2015; Figure 1D). Among the cohort of immature neurons, a subset appear to go through maturation, as demonstrated by the colocalization of DCX with markers of more mature neurons, such as human neuronal protein C and D (HuC/D). More specifically, 1 month following BrdU administration, around 17% of the hypothalamic BrdU+ cells expressed the mature neuronal marker NeuN, confirming the existence of a neurogenic process in the adult sheep hypothalamus (Migaud et al., 2011). In contrasting, in Soay rams, the hypothalamic BrdU-positive cells did not seem to adopt a neuronal phenotype, but rather, 10% of the mitotic cells located in the ME showed morphological features consistent with microglia as they co-express the pan-leukocytic marker CD45 (Hazlerigg et al., 2013). The phenotype of the remaining 90% BrdU positive cells remain unknown. The reason for this discrepancy is not clear, but one hypothesis could be that the maturation process was delayed due to the photoperiodic protocol used, leading to a delayed expression of the marker for mature neurons.

However, in rodent species, the neuronal commitment of the hypothalamic newborn cells was generally verified and between 10 and 37% of the total BrdU-positive cells were found to co-express the neuronal marker. The number of hypothalamic newborn neurons is, therefore, much lower than in the two canonical niches, the SVZ and SGZ. The existence of constitutive hypothalamic neurogenesis at a low rate of neurogenesis was established in rodent models using genetic fate mapping techniques (Lee et al., 2012; Li et al., 2012; Haan et al., 2013; Robins et al., 2013a). By these approaches, the phenotype of the adult born hypothalamic neurons was determined by their expression of peptides relevant for metabolism and feeding control, including neuropeptide-Y (NPY), proopiomelanocortin (POMC; Kokoeva et al., 2005; Li et al., 2012; Haan et al., 2013), and agouti-related protein (AgRP; Pierce and Xu, 2010). In sheep, subsets of hypothalamic neuroblasts also developed specific hypothalamic phenotypes such as NPY (Batailler et al., 2014). In addition, hypothalamic new-born neurons were shown to express estrogen receptor (ER)α, in female mice (Bless et al., 2016) and in ewes (Batailler et al., 2014) indicative of a putative role for these newborn (ER)α cells in the modulation of reproductive behavior (Musatov et al., 2006; Gao and Horvath, 2008).

The sheep hypothalamus appears to be also gliogenic because 70% of the new cells differentiated into S100B+ and GFAP+ astrocytes. However, similarly to what is described in the rat model, no new cells become oligodendrocytes (Steiner et al., 2004). Yet, in mice, Kokoeva et al. (2005) reported the capacity of the hypothalamic adult born cells to produce oligodendrocytes using APC another oligodendrocytic marker. Whether these results reveal difference between species in the time course of glial differentiation requires clarification. Interestingly, adult hypothalamic neurogenesis is notably influenced by various external stimuli, including season (Migaud et al., 2010, 2011, 2015, see below for details), diet (for a review, see Yon et al., 2013), exercise (Niwa et al., 2015) and the social environment (Fowler et al., 2002), which could also explain inconsistencies in the number of newborn neurons detected depending on the study.

#### The hypothalamic neurogenic niche

In contrast with the two main documented niches the SVZ and the SGZ, little is known about the hypothalamic neurogenic niche, and most of our knowledge is inferred from rodent studies. Three cell layers were identified in the adult third ventricle wall: multiciliated cubic ependymal cells, astrocytic subependyma and non-ciliated cells known as tanycytes extending their long cell processes into the hypothalamic parenchyma (Flament-Durand and Brion, 1985). This subset of specialized ependymoglial cells retaining the morphological features of embryonic radial glia cells contact the cerebrospinal fluid. This configuration is generally reminiscent of the generalstructural organization of the SVZ of the lateral ventricle, although the hypothalamic region lined by the tanycytes lacks an identifiable subventricular zone, like the one lining the walls of the lateral ventricles (Doetsch et al., 1997; Alvarez-Buylla et al., 2002).

Four types of hypothalamic tanycytes have been distinguished regarding based on their location within the ventricular wall and gene expression profile (Figure 3). The α1- and α2-tanycytes reside at the level of the ventromedial nuclei and the arcuate nuclei respectively. β1-tanycytes are located in the lateral part of the infundibulum. Finally, β2-tanycytes are positioned in the floor of the third ventricle (Rodriguez et al., 2005). Interestingly, in contrast to other hypothalamic regions, the ME lies outside of the blood-brain barrier, and is therefore a circumventricular organ (Miyata, 2015). Consequently, tanycytes located in the ME are responsive to the hormones, growth factors and nutritional substances transported by the blood and conversely, the tanycytic cell bodies lining the third ventricular wall are exposed to the molecular signals conveyed by the cerebrospinal fluid.

All the tanycyte populations express proteins that are typical for NSC/precursor cells, including Sox2 (Lee et al., 2012; Li et al., 2012), Nestin (Wei et al., 2002), vimentin (Bolborea and Dale, 2013), doublecortin-like protein (DCL; Saaltink et al., 2012), and Alpha-tanycytes express GFAP and the astrocyte-specific glutamate transporter (GLAST) like the SVZ B1 type cells, whereas β tanycytes and a small portion of α tanycytes express Fibroblast growth factor 10 (FGF-10; Li et al., 2012; Robins et al., 2013a), a growth factor implicated in regulating the formation of cortical radial glial cells (Sahara and O'Leary, 2009).

In the sheep hypothalamus, numerous nuclei expressing Sox2 are found in the ependymal or subependymal layers. In addition, almost all cells lining the walls of the third ventricule express the intermediate filament vimentin and most vimentin positive cells, if not all, also strongly coexpressed GFAP in their long processes, producing a staining pattern similar to that previously described in other species, particularly in rodents (Prevot, 2002; Kameda et al., 2003; Baroncini et al., 2007; Langlet et al., 2013). In both the ME and the arcuate nucleus, cells immunoreactive for the three stem cell markers Sox2, GFAP, and Vimentin, are therefore principally distributed in the ependymal and subependymal cell layers lining the third ventricle. The existence of a neurogenic niche in the sheep hypothalamus was further evidenced by the typical tanycyte morphology of the cells lining the third ventricular wall expressing both nestin and Sox2.

The identity of the tanycyte subtypes representing the rodent hypothalamic NSCs is still a matter of debate. Some lineage tracing experiments have revealed that α2-tanycytes have the potential to be the hypothalamic NSCs (Robins et al., 2013a), since the proliferation of α-tanycyte is promoted by FGF2 (Robins et al., 2013a) and the insulin-like growth factor (IGF; Pérez-Martín et al., 2010). In addition, they are the only subtype capable of forming neurospheres and they can give rise to β1-tanycytes. Alternatively, several other studies using different lineage tracing mice models have shown that β-tanycytes are the major sources of progenitor cells in the hypothalamus of young adult mice (Bolborea and Dale, 2013; Haan et al., 2013). More specifically, β2 cells are highly proliferative and neurogenic in young animals (Lee et al., 2012, 2014). Due to our large mammalian model, it is hardly conceivable to develop such intersectional genetic approaches in order to identify the tanycyte(s) subtypes incriminated. However, the recent introduction of the “CRISPR-cas9” technology (Hsu et al., 2014) and the potential regulatory programmable schemes employed in the future to provide temporal control for these reagents (Guha et al., 2017) will be useful to modify the genome, regardless of the mammalian species. These strategies will undoubtedly participate in our understanding of neurogenesis mechanisms in large mammalian species like sheep.

While tanycytes surely constitute a major class of NSCs of the hypothalamic neurogenic niche, a subset of Sox2 expressing cells also showing a proliferative activity has been detected in the hypothalamic parenchyma in sheep (Migaud et al., 2011; Batailler et al., 2014) and in rodents (Kokoeva et al., 2005; Li et al., 2012; Robins et al., 2013b). Whether these cells contribute to the neurogenic process and to hypothalamic physiological functions is currently unknown.

In sheep, numerous DCX-positive immature neurons are observed in the arcuate nucleus, likely originating from the germinative zones lining the ventral part of the third ventricle (Batailler et al., 2014). The morphology of the DCX-positive cells changes whether they are close to or distant from the third ventricle cavity. DCX-positive cells near the third ventricle show a rather round shape with no processes, typical of very immature neuroblasts. Conversely, DCX-positive cells found in the parenchyma of the ventromedial hypothalamus show fusiform perikarya and lengthened processes, some of the characteristics of migrating cells undergoing maturation (Figure 3; Batailler et al., 2014). One hypothesis is that neuroblasts located in the subependymal niche may spread toward neighboring hypothalamic nuclei and form a migratory path (Batailler et al., 2014), as it has been suggested in rodents (Haan et al., 2013). One way to test this hypothesis would be to label progenitors with iron oxide particles and detect their putative migration by means of magnetic resonance imaging.

In contrast, a very different pattern of DCX labeling was found in the mouse hypothalamus (Kokoeva et al., 2007; Batailler et al., 2014), with only low-intensity labeled cells detected in the arcuate nucleus and moderate to high levels of labeled fibers in the ME. This was true in both sexes, excluding an effect of the estrous cycle. Likely differences in the function of these DCX-positive cells between species may be the reason for this discrepancy.

The flow of adult rodent hypothalamic neurogenesis is promoted by numerous growth factors, including IGF-1 (Pérez-Martín et al., 2010; Chaker et al., 2016), BDNF (Pencea et al., 2001), ciliary neurotrophic factor (CNTF; Kokoeva et al., 2005), FGF, and epidermal growth factor (Xu et al., 2005; Pierce and Xu, 2010, reviewed in Sousa-Ferreira et al., 2014). Additionally, a recent study also reported an enhancement of the hypothalamic proliferating activity in aged mice by gonadotropin releasing hormone (Zhang et al., 2013). As with the DG and the SVZ, the rate of hypothalamic proliferation may be affected by exogenous factors. For example, in the highly social prairie vole male exposure enhances cell proliferation in the female hypothalamus and the amygdala when compared to socially isolated animals (Fowler et al., 2002). Voluntary exercise may also enhance hypothalamic cell turnover in rats (Niwa et al., 2015).

## Physiological regulation of neurogenesis

### Seasonal regulation

In seasonal species, photoperiod is a critical environmental cue required for the seasonal programming of reproductive and metabolic functions, an adaptive strategy to cope with the annual fluctuations in climate, temperature, and food availability. The influence of seasons and photoperiod on adult cell proliferation and neurogenesis, mainly in the two canonical niches, has been extensively studied in a broad range of mammalian seasonal species including Golden (Huang et al., 1998) and F1B (Smith et al., 2001) hamsters, deer mice (Perrot-Sinal et al., 1998), white footed mice (Walton et al., 2012), meadow voles (Galea and McEwen, 1999; Galea et al., 1999; Ormerod and Galea, 2003), Richardson's ground squirrel (Burger et al., 2013), eastern gray squirrels (Lavenex et al., 2000), shrew (Bartkowska et al., 2008), and sheep (Migaud et al., 2010, 2011; Hazlerigg et al., 2013).

Seasonal changes in cell proliferation have been detected in the DG of adult female meadow voles (Galea and McEwen, 1999; Galea et al., 1999). In this species, females show higher levels of cell proliferation than males and higher levels during the non-breeding than during the breeding period (Galea and McEwen, 1999; Ormerod and Galea, 2003). In the DG of the golden hamster, a two-fold increase in the number of dividing cells has been found after a transition from long days to short days (Huang et al., 1998). Photoperiod also affects cell proliferation in DG of the Richardson's ground squirrel (Burger et al., 2013), the Soay ram (Hazlerigg et al., 2013), and the shrew (Bartkowska et al., 2008). In contrast, some studies failed to detect cell proliferation changes in male meadow voles (Ormerod and Galea, 2003) or in squirrels (Lavenex et al., 2000).

The olfactory neurogenic niche is also influenced by photoperiodic changes. In short day exposure, Syrian hamsters show an increased rate of neurogenesis in the MOB (Huang et al., 1998). In white-footed mice, an increase in new neurons in the caudal olfactory bulb resulting from short day exposure is linked with changes in behavioral responses to the urine of conspecific males (Walton et al., 2012), suggesting that olfactory neurogenesis may be a mechanism underlying photoperiodic variations in social interactions. In contrast, in two species of photoperiodic shrews, short day exposure induces a reduction in SVZ proliferation and neurogenesis (Bartkowska et al., 2008). In the sheep SVZ, no cell proliferation changes are observed across the seasons (Migaud et al., 2011). All these data indicate species-specific effects of the photoperiod on the neurogenesis in the SGZ and the SVZ.

By contrast, only a few studies have examined the influence of season on adult hypothalamic neurogenesis. In Syrian hamsters, a transition from long to short days increases cell proliferation (Huang et al., 1998). In Siberian hamster tanycytes, the expression of the transcript for nestin, an intermediate filament protein used as a neural stem cell marker (Kronenberg et al., 2003; for review see Wiese et al., 2004), is down-regulated during short-day photoperiod exposure (Barrett et al., 2006; Ebling and Barrett, 2008).

In Ile-de-France ewes, the proliferative capacity of the hypothalamus is seasonally regulated (Migaud et al., 2010, 2011). Significantly more new hypothalamic cells are generated, independent of sex steroids, during the short days (corresponding to the period of sexual activity in this species) compared with the long days (coinciding with the period of sexual inactivity; Migaud et al., 2011). However, in Soay rams, no clear seasonal variation is observed in the level of cell proliferation, whatever the hypothalamic region considered (Hazlerigg et al., 2013). The reason for this discrepancy is unknown but might depend on differences in the immunohistochemical procedure, or in the photoperiodic treatment provided. Nevertheless, a higher density of DCX-expressing neurons is found in the arcuate nucleus and the ME (Batailler et al., 2014), the most neurogenic region of the hypothalamus (Lee et al., 2013), during the stimulatory short photoperiod compared with the inhibitory long photoperiod. These data suggest that seasonal regulation of neurogenesis might be a common regulatory mechanism among adult seasonal mammals.

### Regulation by physiological status and social interactions

The different processes of neurogenesis, mainly production, maturation, and survival are also under the control of various internal factors (Lledo et al., 2006). Many studies have shown that the estrous cycle, pregnancy, and parturition regulate hippocampal and olfactory neurogenesis in laboratory rodents (for review: Pawluski et al., 2009; Lévy et al., 2011). In sheep, physiological changes associated with parturition influence cell proliferation in the DG and the MOB. Mothers permanently separated from their newborn lambs immediately after parturition show a decrease in cell proliferation, evidenced by BrdU labeling, in the MOB and in the sub-granular zone of the DG compared to non-separated ewes (Brus et al., 2010). In a follow-up study, using Ki67 as a marker of proliferation, a similar decrease in proliferation was found in both the MOB and the DG of parturient ewes in comparison to non-pregnant females (Brus et al., 2014).

This down-regulation could be the consequence of the change in circulating steroids occurring at parturition. In the female rat, a regime of ovarian steroids that mimicks the fluctuations occurring at birth, decreases cell proliferation in the DG (Tanapat et al., 2005; Pawluski et al., 2009). The increased cortisol levels at parturition also decreases cell proliferation in the DG (Tanapat et al., 1999; Darnaudery et al., 2007). In sheep, both oestradiol and cortisol could be involved in the decreased cell proliferation in the DG and in the MOB. Interestingly, estrogen and glucocorticoid receptors are found in both structures (Morimoto et al., 1996; Shughrue et al., 1997; Sah et al., 2005). This down-regulation is not observed in the SVZ, suggesting that olfactory cell proliferation could be differently regulated according to the brain region. Supporting this view, the SVZ lacks estrogen and glucocorticoid receptors (Shughrue et al., 1997; Shughrue and Merchenthaler, 2000) and cortisol treatment differentially affects the DG and the SVZ, reducing neural production in the DG but sparing it in the SVZ (Siopi et al., 2016).

Regulation of cell survival by parturition differs from that of cell proliferation because changes in survival are found only for the DG but not for the MOB. Survival is reduced by parturition as indicated by the finding that mothers separated from their young at parturition show a down-regulation of BrdU+/NeuN+ cells (Brus et al., 2014). Cortisol release at parturition could be involved, as well as other endocrine factors as oxytocin (Leuner et al., 2012). By contrast, no change in the number of neuroblasts is found in these parturient ewes, suggesting differential regulation according to the maturity stage, which has been reported for the effects of corticosterone in the DG of mice (Gonzalez-Perez et al., 2011; Lussier et al., 2013).

Not only can endocrine changes regulate neurogenesis, but social factors can also affect cell proliferation and survival (Gheusi et al., 2009; Holmes, 2016). In sheep, a reproductive cycle can be induced in anoestrus females when exposed to males (Martin et al., 1986), which doubles the rate of cell proliferation in the DG (Hawken et al., 2009). In addition, in sheep maternal behavior at parturition depends on olfactory attraction toward amniotic fluids that cover the newborn lamb (Lévy et al., 2004; Poindron et al., 2007; Lévy and Keller, 2009). These cues render the newborn lamb attractive and stimulate its licking by the mother, thus inducing maternal behavior. Moreover, ewes are able to discriminate their own young from an alien lamb by learning its olfactory signature within 2 h after parturition (Lévy et al., 2004; Lévy and Keller, 2009). This learning is accompanied with neurochemical changes occurring in the MOB (Lévy et al., 1993; Lévy and Keller, 2009). Olfactory neurogenesis could also contribute to the onset of maternal behavior and associated learning, and altered neurogenesis during the establishment of maternal behavior support this hypothesis. Brus et al. (2010) show that decreased cell proliferation occurs in the SVZ, but not in the DG, in ewes that remain with their lambs for the first 2 days after parturition when compared to ewes separated from them. However, SVZ cell proliferation is not affected in ewes mating with a male, and thus appears to be specific to interactions with the young. Consistent with these findings, the survival of neuroblasts in the MOB is also reduced in ewes interacting with their lamb but maturation of the remaining neuroblasts is heightened (Brus et al., 2014). Interactions with young and associated olfactory learning rather than parturition are responsible for these modifications because they are prevented by separating ewes from their lambs at parturition. Numerous studies report that olfactory experience sculpts newborn neurons (for review: Lazarini and Lledo, 2011), with nostril closure decreasing (Saghatelyan et al., 2005) and odor enrichment increasing the arborization complexity of newborn granule cells (Livneh and Mizrahi, 2011). In the context of motherhood, olfactory exposure to pups induces changes in structural synaptic plasticity of newly born olfactory neurons in mice (Kopel et al., 2012; Belnoue et al., 2016). Although, the functional relevance of the plasticity occurring in the MOB of sheep remains to be determined, one can hypothesize that the decrease in the number of neuroblasts would reduce cell competition and consequently increases their maturation, allowing them to be integrated in the neural network involved in learning. In support to this proposition, neural network models of hippocampal neurogenesis show that high levels of cell proliferation are have negative effects on the stability of neural activity and consequently for learning (Lehmann et al., 2005; Butz et al., 2006). Another modeling study indicates that an increase in cell proliferation causes a reduction in the amount of synaptic rewiring which is not beneficial for learning (Butz et al., 2008). Whether these findings can be applied for olfactory neurogenesis remains to be determined.

## Functional relevance of neurogenesis

### Hypothalamic neurogenesis

The hypothalamus is an essential homeostatic regulator of many physiological and behavioral processes, such as reproduction, feeding, growth, metabolism, body temperature, and circadian rhythms (Saper and Lowell, 2014). Some of the important roles of this structure are to integrate sensory inputs with hormonal and peripheral signals, to control pituitary hormone secretions and regulate the downstream major biological functions. Evidence for adult hypothalamic neurogenesis raises the issue of its functional role that is still in its early exploratory phases. Because of the key role plays by the hypothalamus in metabolism and food intake, the significance of hypothalamic neurogenesis in these functions has been the most explored so far. In mice, hypothalamic newborn neurons acquire the identities and the functional phenotypes related to the control of energy homeostasis, including NPY or POMC (Kokoeva et al., 2005; McNay et al., 2012; Gouaze et al., 2013). In addition, some of these new neurons are responsive to fasting and leptin (Kokoeva et al., 2005; Pierce and Xu, 2010; Haan et al., 2013). Several recent studies show that diet can regulate adult hypothalamic neurogenesis, although the results are equivocal. Opposing effects of high fat diet on neurogenesis and body weight are reported depending on the ages and sexes of the animals tested, as well as the duration of the diet and the targeted hypothalamic area (Lee et al., 2012; Li et al., 2012). In mice, the loss of weight induced by the administration of the CNTF is suppressed when administered concomitantly with the cytosine arabinoside (Ara-C), an antimitotic drug, which blocks hypothalamic cell proliferation indicating a role of hypothalamic neurogenesis in weight regulation (Kokoeva et al., 2005).

The hypothalamus is also the integrative center that regulates reproduction, and newly formed hypothalamic cells express ERα (Batailler et al., 2014; Bless et al., 2016), a receptor involved in many aspects of both male and female reproduction (Ogawa et al., 1998; Emmen and Korach, 2003). In sheep, to explore the role of hypothalamic neurogenesis in the seasonal control of reproductive function, Ara-C was administered into the third ventricle for 30 days during the peak of hypothalamic cell proliferation (Batailler et al., submitted). This treatment induced a notable 75% decrease in hypothalamic neurogenesis assessed by the density of DCX-positive cells. This decrease resulted in an advance of the entry in seasonal anoestrus and a similar advance in the re-entry in sexual activity the following season. Interestingly, in our experimental conditions, none of the animals showed altered body weight. These data draw a strong interaction between new neuron production and the seasonal adaptation of neuroendocrine networks, with hypothalamic neurogenesis being likely involved in the functional adaptation of the brain to the changing environmental conditions. The precise identification of the phenotype and the destinations of the newborn neurons will give insights to the molecular pathways involved in these processes.

### Olfactory neurogenesis

In mammals, only a few studies have looked at changes in social behavior induced by reducing or blocking neurogenesis (Holmes, 2016). In sheep, the functional relevance of neurogenesis has been examined in the context of maternal behavior by testing the hypothesis that increased activation of newborn neurons after exposure to lambs is behaviourally relevant. By pairing neurogenic markers with markers of neural activity, activation of olfactory newborn neurons have been compared between mothers exposed either to their own lamb, an unfamiliar lamb, or to an adult conspecific (Corona et al., 2016). Exposure to either both lambs increases the percentage of neuroblasts activated in the granular layer of the MOB compared to exposure to an unfamiliar ewe, indicating that the preferential activation is not seen for any social odors but is specific to lamb odors. By contrast, newborn neurons in the DG do not show any activation in response to any of the odors.

Hence, newborn neurons of the MOB could participate to the processing of olfactory cues responsible for maternal attraction to any newborn lambs at parturition. However, these olfactory adult-born neurons fail to differentiate between familiar and unfamiliar lamb exposure, so another pool of newborn neurons of distinct ages could be involved in recognition of the familiar lamb.

To further understand the functional relevance of olfactory neurogenesis disruption of neurogenesis was performed and its consequences on maternal behavior was assessed. To prevent adult olfactory neurogenesis in parturient sheep mothers, infusion of Ara-C into the SVZ for 1 month during pregnancy was performed and the consequences on maternal behavior and recognition of the familiar lamb assessed during early postpartum period (Corona et al., submitted). Ara-C infusion led to a 70% reduction in olfactory neurogenesis, evidenced by DCX labeling, sparing hippocampal neurogenesis. The impairment of olfactory neurogenesis was found to reduce maternal vocalizations in the presence of the familiar lamb at parturition and during selectivity tests. However, all the ewes are maternal and selective for their own lamb. These relatively minor effects on mothering could be related to limitations of the method used for blocking neurogenesis. Although the levels of olfactory neurogenesis are dramatically reduced by Ara-C infusion, it is possible that the remaining olfactory newborn neurons are sufficient to sustain maternal behavior and olfactory recognition of the familiar lamb. Similarly, in mice infusion of an anti-mitotic agent into the SVZ induces little disturbance of maternal behavior (Larsen and Grattan, 2010) and irradiation of the SVZ does not alter either maternal behavior or dams' ability to discriminate familiar pups from alien pups (Feierstein et al., 2010). However, genetic manipulations inducing continuous inhibition of neurogenesis during pregnancy and postpartum disrupts nursing (Sakamoto et al., 2011). Therefore, a more extended ablation of olfactory neurogenesis would be helpful to assess the role of olfactory neurogenesis in motherhood of sheep.

## Conclusion and future directions

The generation of new neurons in the adult mammalian brain, long thought to be non-existent, is now widely established although the issue of human olfactory neurogenesis is still under debate (Curtis et al., 2007; Bergmann et al., 2012). Over the last few decades, studies performed in rodents have led to considerable improvements in our understanding of this phenomenon. However, as shown in the present review, the cellular and molecular machinery and the functional mechanisms behind how this process influences adult brain circuits may differ qualitatively and quantitatively between species according to their brain features, lifespan, physiological, and behavioral burdens (Table 1). These differences between species stress the importance of analyzing the neurogenic process in multiple different model systems. Overall, this review highlights sheep as an appropriate model to investigate the role of adult neurogenesis during individual recognition/discrimination and in neuroendocrine behaviors related to reproduction, although further experiments are needed to explore in depth the neural circuits underlying these relationships as well as the physiological underpinnings involved.

**Table 1:** Comparative organization of the three neurogenic niches the SVZ, SGZ, and the hypothalamic neurogenic niche (Hyp) in five mammalian species.

	**SVZ**					**SGZ**		**Hyp**		
	**PPCs**	**Hypocellular Gap**	**RMS**	**Migration rates**	**Neurogenic olfactory ventricle**	**PPCs**	**Adult born GCs**	**PPCs**	**DCX+ Neuroblasts**	**Adult born neurons**
Rodents	++	0	+	3–7 days in mice	0	++	+	++	±	POMC, NPY, AgRP
				10–12 days in rats						
Rabbit	++	+	+	ND	+	ND	ND	ND	ND	ND
NHP	++	+	+	2–3 months	0	++	+	ND	ND	ND
Sheep	++	+	+	1 month	+	+	+	++	++	NPY
Adult Human	+	0	0	ND	0	++	++	ND	ND	ND

*NHP, Non-human primates; PPCs, proliferative progenitor cells; RMS, rostral migratory stream; ++, strong presence; +, presence; ±, weak presence; 0, absence; ND, Not determined. POMC, Pro-Opiomelanocortin. NPY, Neuropeptide Y; AgRP, Agouti related Peptide. Rodents, Lois and Alvarez-Buylla, 1994; Kokoeva et al., 2005; Pierce and Xu, 2010; Lee et al., 2012; Haan et al., 2013; Rabbits, Bonfanti and Ponti, 2008; Larriva-Sahd, 2014; NHP, Gil-Perotin et al., 2009; Sawamoto et al., 2011; Wang et al., 2011; Sheep, Migaud et al., 2011; Brus et al., 2013; Batailler et al., 2014. Adult human, Quinones-Hinojosa et al., 2006; Butz et al., 2008; Sanai et al., 2011; Spalding et al., 2013*.

Similar to laboratory rodents, studies performed in sheep have demonstrated the existence of neurogenic niches in the SVZ, the SGZ, and the hypothalamus. In contrast, the cellular composition and the morphological organization of the sheep SVZ and the hypothalamus differ from that of rodents (Table 1). Furthermore, a longer maturation time frame is observed for newborn neurons in sheep compared with rodents. These latter features appear to share similarities with the non-human primate brain. In addition, a comparable pattern of distribution of neuroblasts is reported in sheep and human hypothalamus, but both different from the murine hypothalamus (Batailler et al., 2014). Thus, sheep could be a suitable alternative model to primates to gain insights into the function of adult neurogenesis for therapeutics in humans. Currently, no data are available on the time required for the full neuronal maturation of the newborn neurons in our species and further investigation is necessary to compare the timing of differentiation of the new neurons in humans and in non-human primates or sheep. Increasing our knowledge on the existence of newly formed neurons in humans will also require the development of advanced imaging techniques. In some regards, sheep are already considered as an important developing model for translational imaging procedures (Forschler et al., 2007; van der Bom et al., 2013; Beuing et al., 2014) and in support of that the ovine brain template and corresponding tissue probability maps have very recently been generated and made available (Ella and Keller, 2015; Nitzsche et al., 2015). In our sheep, a magnetic resonance imaging study is currently being undertaken to label progenitors with iron oxide particles to detect their migration. The implementation of neuroimaging procedures enabling the study of neurogenesis in an appropriate animal model, such as sheep, will very likely lead to the development of translational experiments performed in normal and pathological human brains.

## Author contributions

FL, MB, MMe, and MMi wrote and edited the manuscript. MB and MMe drew the figures.

### Conflict of interest statement

The authors declare that the research was conducted in the absence of any commercial or financial relationships that could be construed as a potential conflict of interest.
